# Physiological insights into sulfate and selenium interaction to improve drought tolerance in mung bean

**DOI:** 10.1007/s12298-021-00992-6

**Published:** 2021-05-04

**Authors:** Muhammad Aqib, Fahim Nawaz, Sadia Majeed, Abdul Ghaffar, Khawaja Shafique Ahmad, Muhammad Asif Shehzad, Muhammad Naeem Tahir, Muhammad Aurangzaib, Hafiz Muhammad Rashad Javeed, Muhammad Habib-ur-Rahman, Muhammad Munir Usmani

**Affiliations:** 1 Department of Agronomy, MNS University of Agriculture, Multan, Pakistan; 2 Institute of Crop Science (340 h), University of Hohenheim, Stuttgart, Germany; 3 Department of Agronomy, The Islamia University of Bahawalpur, Bahawalpur, Pakistan; 4 Department of Botany, University of Poonch, Rawalakot, 12350 Pakistan; 5 University College of Veterinary and Animal Sciences, The Islamia University of Bahawalpur, Bahawalpur, Pakistan; 6 Department of Environmental Sciences, COMSATS University, Vehari Campus, Vehari, Pakistan; 7 Institute of Crop Science and Resource Conservation (INRES) Crop Science, University Bonn, Bonn, Germany; 8 Present Address: Alexander von Humboldt Postdoctoral Fellow at University of Hohenheim (340 h), 70599 Stuttgart, Germany

**Keywords:** Sulfate, Selenate, Photosynthetic apparatus, Mineral content, Drought, *Vigna radiata*

## Abstract

The present study involved two pot experiments to investigate the response of mung bean to the individual or combined SO_4_^2−^ and selenate application under drought stress. A marked increment in biomass and NPK accumulation was recorded in mung bean seedlings fertilized with various SO_4_^2−^ sources, except for CuSO_4_. Compared to other SO_4_^2−^ fertilizers, ZnSO_4_ application resulted in the highest increase in growth attributes and shoot nutrient content. Further, the combined S and Se application (S + Se) significantly enhanced relative water content (16%), SPAD value (72%), photosynthetic rate (80%) and activities of catalase (79%), guaiacol peroxidase (53%) and superoxide dismutase (58%) in the leaves of water-stressed mung bean plants. Consequently, the grain yield of mung bean was markedly increased by 105% under water stress conditions. Furthermore, S + Se application considerably increased the concentrations of P (47%), K (75%), S (80%), Zn (160%), and Fe (15%) in mung bean seeds under drought stress conditions. These findings indicate that S + Se application potentially increases the nutritional quality of grain legumes by stimulating photosynthetic apparatus and antioxidative machinery under water deficit conditions. Our results could provide the basis for further experiments on cross-talk between S and Se regulatory pathways to improve the nutritional quality of food crops.

**Supplementary Information:**

The online version contains supplementary material available at 10.1007/s12298-021-00992-6.

## Introduction

Climate change has increased the vulnerability of cropping systems, which may trigger a loss of livelihoods of poor communities in arid and semi-arid regions of the world (Leng and Hall 2019). The imbalance in the temporal and spatial distribution of rainfall and the non-availability of the required amount of water during critical growth stages would seriously limit the productivity of major food crops in near future (Troy et al. 2015). The non-availability of water severely hampers crop production in developing and arid regions of the world where people are solely dependent on rainwater for their survival. Detrimental effects of drought are likely to aggravate the world’s limiting available fresh water supply to fulfill the food demand for the ever-increasing population (Leng 2017). In this regard, it is imperative to recognize the importance of climate-resilient crops such as legumes and promote their value throughout the food systems.

Legumes are an integral part of a healthy and balanced human diet and play important role in preventing many acute diseases. Moreover, due to their atmospheric nitrogen (N) fixing ability, they increase soil C and N, reduce soil erosion and help to control soil pathogens (Rubiales and Mikic 2015). Mung bean (*Vigna radiata* (L.) R. Wilczek) is an important food legume that is grown primarily for dry seeds (Kudre et al. 2013). Although, mung bean is considered tolerant to limited water supply, low water availability at reproductive and grain filling stages significantly reduces its yield and quality (Gaur et al. 2012). Drought-induced changes in mung bean include reduced carbon fixation, repressed flowering time, increased pollen sterility, fewer pods, and poor seed set (Nadeem et al. 2019). Legume crops like mung bean are commonly cultivated in rainfed regions; hence, development of sustainable mitigation approaches that could easily integrate with farming practices of dry areas is essential to ensure high yield under harsh environment (Raina et al. 2016). Application of mineral nutrients actively involved in stress tolerance and defense mechanisms could be used as a viable option to alleviate the damaging effects of environmental stresses in legumes (Afzal et al. 2015).

Sulfur (S) is an important element that plays a crucial role to mitigate abiotic stresses in crop plants. Increased sulfate (SO_4_^2−^) demand during metabolic adaptation to drought reflects its importance in the regulation of plant defense machinery (Usmani et al. 2020). Drought stress increases the concentration of SO_4_^2−^ compared to other ions like phosphate or nitrate, providing evidence that sulfate demand increases under limited water conditions (Ernst et al. 2010). SO_4_^2−^ interacts with abscisic acid (ABA) and acts as a chemical signal to initiate stomatal closure in leaves under water deficit conditions. Metabolism of S results in the formation of several S-containing defense compounds such as methionine, cysteine, glutathione, and phytochelatins that are involved in plant survival during various abiotic stresses including drought (Honsel et al. 2012). The interplay of S with phytohormones helps to regulate crucial metabolic processes in plants (Noctor et al. 2012). Deficiency of S may result in impaired growth, loss in plant production, and susceptibility to environmental stresses due to reduced protein synthesis, chlorophyll degradation and decreased photosynthetic rate (Fatma et al. 2016).

Akin to S, selenium (Se) is an important micronutrient considered essential for animals and required in very minute quantities by plants. Both Se and S share similar chemical properties and are taken up by plants through sulfate transporters and assimilated by S assimilating pathway (Sors et al. 2005). In most countries, the major dietary source of Se is plant food (Rayman 2008). Se deficiency in the human diet causes abnormalities in thyroid function alters bone metabolism, and leads to retarded growth (Reeves and Hoffman 2009). In Se deficient areas, exogenous application with Se or Se-enriched compounds can be used as an efficient, cost-effective and viable approach to enrich plants with Se (Sabatino et al. 2019). Moreover, Se application stimulates growth, improves physiological processes (Bocchini et al. 2018), and enhances tolerance against various environmental stresses (Banerjee and Roychoudhury 2019). It is considered as a constituent of selenoenzymes (thioredoxin reductases, glutathione peroxidase, and proteins), which are vital to maintaining cell redox potential (Rayman 2008). Se increases plant growth (Nawaz et al. 2013), carbohydrate accumulation (Kaur et al. 2018), and delays senescence in crop plants (Hajiboland et al. 2019). The positive effects of Se to mitigate biotic and abiotic stresses including UV-B radiation (Golob et al. 2017), cadmium toxicity (Shahid et al. 2019), fungal infections (Kornaś et al. 2019), and drought (Nawaz et al. 2015a) are well documented.

To date, only few studies have been conducted to investigate the yield changes in leguminous crops like mung bean by Se supply under drought stress. This study partially fills the gap by determining the effects of Se application and its interrelation with S in mung bean exposed to water deficit conditions. The study aimed to i) evaluate the effects of Se or S nutrition on physiological mechanisms of mung bean under drought stress and ii) assess whether combined application of Se and S is more effective than individual treatment to improve yield and mineral content of mung bean under water deficit conditions.

## Materials and Methods

### Experimental conditions and material

The experiments were conducted in two phases: initially, the locally cultivated mung bean cultivars were screened for their response to various S fertilizers in the first experiment. Later in the second experiment, the most responsive mung bean cultivar was evaluated for its response to individual or combined S and Se doses under normal and water-deficient conditions. The experiments were performed in a green house (C-block, MNS-University of Agriculture, Multan, Pakistan) with ambient temperature (average day/night: 36/23 °C) and light (photoperiod: 14/10 h) conditions during the growth period. A manually controlled moveable polythene sheet was installed to protect the plants from rainwater. The experimental design was completely randomized design (CRD) with the factorial arrangement and three replications (03 plants per replication/pot were maintained till final harvest). The pot experiment-I comprised of 45 pots, whereas 36 pots were used in the pot-experiment-II. The pots were filled with sun-dried, grounded, and thoroughly mixed clayey-loamy soil collected from the farm area of the University located at B-block. The physicochemical characteristics of the soil were determined following the reports of Jackson and Barak (2005) and given in Table 1.

**Table 1:** Physico-chemical characteristics of soil used in the pots for the experiments

Texture	Soil moisture content (%)	EC (mScm^−1^)	pH	OM (%)	N (%)	P (mg kg^−1^)	K (mg kg^−1^)	S (%)
Loamy sand	11.2	2.47	7.9	0.74	0.076	8.50	240	0.032

### Pot experiment-I

The first experiment was conducted with the objective to evaluate the response of mung bean cultivars to various S-sources. The experiment was initiated on May 06, 2018, for four weeks. Seeds of three local mung bean cultivars viz. NM-2006, NM-2011, and AZRI-2006 were obtained from Punjab Seed Corporation, Faisalabad (Pakistan). Initially, six healthy and physically pure seeds were sown in pots with 15 L capacity (height 25 cm and diameter 30 cm), and each filled with 12 kg clayey-loamy soil. After the establishment of seedlings, thinning was done to maintain three healthy plants per pot. Before sowing, recommended doses of fertilizers such as urea (25 kg ha^−1^, 150 mg pot^−1^) and diammonium phosphate (50 kg ha^−1^, 300 mg pot^−1^), and potassium hydroxide (50 kg ha^−1^, 300 mg pot^−1^) were added as nutrient solutions to meet the nutritional requirement of the plants in each pot. To ensure that each pot received an equal amount of S (20 kg ha^−1^, 120 mg pot^−1^), different fertilizer treatments of zinc sulfate (ZnSO_4_, 1090 mg pot^−1^), iron sulfate (FeSO_4_, 571 mg pot^−1^), potassium sulfate (K_2_SO_4_, 667 mg pot^−1^), and copper sulfate (CuSO_4_, 923 mg pot^−1^) were added through fertigation in each pot. After four weeks, the seedlings were harvested to determine the effects of S fertilization on biomass accumulation and nutrient uptake in different mung bean cultivars. The seedlings were washed thoroughly to avoid any sand particles and the samples were packed in plastic bags and tagged according to the treatments.

### Pot-experiment II

In this experiment, the role of S and/or Se nutrition to improve the growth and yield of mung bean under drought stress was investigated. The best performing mung bean cultivar i.e. AZRI-2006, selected from pot-experiment-I, was evaluated for its response to individual or combined S and Se nutrition. The S-fertilizer (ZnSO_4_, 1090 mg pot^−1^) was selected based on results of pot-experiment I and was evaluated alone or in combination with two Se doses i.e. Se_1_ (2 μmol Se L^−1^) and Se_2_ (4 μmol Se L^−1^) using sodium selenate (Na_2_SeO_4_) as a source of Se. The experiment was conducted during the growth period of 2018 (June 14 to September 20). Physically pure and healthy seeds of selected mung bean cultivar (AZRI-2006) were sown in brick pots, as described in pot-experiment-I. The nutritional requirements in the form of N, P, and K along with ZnSO_4_ were fulfilled at the start of the experiment (two days before sowing) as described earlier, whereas Se treatments as foliar spray were done one week after the onset of drought.

### Drought stress and foliar Se treatment

All pots were watered regularly considering the moisture requirements calculated using the method reported by Giriappa (1988). The seedlings were allowed to establish under normal conditions for the first two weeks and then drought stress was initiated by withholding water supply for the next 15 days to one set of pots (drought stress) compared to normal plants watered at regular intervals. Foliar spray of Se was done one week after the initiation of drought using compression sprayer as described by Nawaz et al. (2015a) and repeated the next week on the same day. The spraying was done manually in the early hours of the morning (between 8:00 and 9:00 a.m.) and each treatment solution was added with Tween-20 (0.1%) to enhance the adhesion of Se to foliage. Data regarding gas exchange characteristics was recorded two days after the second spray, whereas the leaf samples for physiological and biochemical analysis were collected the very next day. The plants were grown till maturity and harvested manually at physiological maturity when about more than 80% of pods turned yellow.

### Morphological attributes and shoot NPK content

The seedlings were manually uprooted to collect data regarding biomass accumulation. The soil around the seedlings was dug deep and seedlings were removed with extreme care to minimize damage to the roots. A metering rod was used for the measurement of the root length (RL) of each seedling. Observed data from selected plants were recorded in cm (centimeters) from the basal end of the root to the bottom of the shoot and the mean of three readings was calculated. The shoot length (SL) of above-ground part of each seedling was calculated in centimeters (cm) using a meter rod. The seedlings were then weighed immediately to record shoot fresh weight (SFW) in grams (g) using a digital balance. The shoots were then placed in an oven at 65 °C for 72 h to record shoot dry weight (SDW).

The oven-dried shoot samples (above ground material) were grounded and later these finely ground shoot samples (0.5 g) were acid digested using 5 ml HNO_3_ and 2 ml H_2_O_2_ at 350 °C for three hours in digestion block. This was done until the solution becomes colorless. After digestion, the solution was cooled to room temperature and distilled water was added to make the final volume up to 50 ml. The shoot N content was determined by Kjeldahl method (AOAC 1984), whereas the Vanadium molybdate yellow calorimetric method (Chen et al. 1956), and Flame photometry (Karpiuk et al. 2016) were employed to estimate P and K contents in shoots, respectively.

### Estimation of leaf water status and SPAD value

The leaf water status of normal and drought-stressed mung bean plants was estimated as leaf relative water content (RWC) using leaf samples (0.5 g) from the uppermost fully expanded leaf (Schonfeld et al. 1988). The leaves were weighed immediately (FW), re-hydrated in distilled water for 24 h in the refrigerator (4 °C) to record turgid weight (TW), and later dried in an oven at 70 °C until constant weight (DW) was obtained. Leaf RWC was calculated as 100 x (FW-TW)/(FW-DW).

Randomly selected top five fully expanded leaves were selected in each pot to measure leaf total chlorophyll content (Chl) using SPAD meter (SPAD-502 plus, Konica-Minolta, Beijing, China). The readings were used to calculate the average SPAD value for each treatment.

### Measurement of leaf gas exchange characteristics

The leaf gas exchange characteristics viz. photosynthetic rate (*A*), stomatal conductance (*g*_*s*_), sub-stomatal conductance (*C*_*i*_), and transpiration rate (*E*) were recorded from the second fully expanded young mature leaf from the top using portable photosynthesis system CIRAS-3 (PP Systems, Amesbury, U.S.A.). These observations were made in the morning between 8:00 and 10:00 with the adjustments as reported in Shehzad et al. (2020).

### Antioxidative enzymes assay

Fresh leaf samples (0.5 g) were homogenized in an ice-cold mortar and pestle (1:5 w/v) using a mixture of sodium phosphate buffer (50 mM, pH 7.0), polyvinylpyrrolidone (1%), EDTA (1 mM), and NaCl (1%). The crude extract was centrifuged at 20,000xg for 15 min and the supernatant, denoted as enzyme extract (EE), was used to determine the antioxidant enzyme activity.

Catalase (CAT) activity was determined following the procedure of Aebi (1984). Briefly, 200 μL EE was added to 1.8 mL RM (reaction mixture) prepared by mixing K-P-buffer (50 mM, pH 7.0) and H_2_O_2_ (30 mM). The degradation of H_2_O_2_ was recorded as a reduction in optical density at 240 nm.

The method published by Urbanek et al. (1991) was followed to determine the leaf guaiacol peroxidase (GPX) activity. Initially, 25 μL EE was added to 2 ml solution prepared by mixing K-P-buffer (50 mM, pH 6.8), H_2_O_2_ (20 mM), and guaiacol (20 mM). After incubation at room temperature for 10 min, the reaction was stopped by adding 0.5 mL H_2_SO_4_ (5% v/v) and the absorbance was noted at 480 nm.

The enzyme activity of superoxide dismutase (SOD) was assayed by adding 50 μL EE in RM containing L-methionine (13 mM), NBT or nitro blue tetrazolium chloride (75 μM), riboflavin (2 μM)) and EDTA (100 μM) in K-P-buffer (50 mM, pH 7.8). The reaction was initiated under the illumination of a 30 W-fluorescent lamp for 5 min, resulting in the formation of blue formazan measured as an increase in absorbance at 560 nm using RM (kept in the dark) as a blank (Van Rossum et al. 1997).

### Determination of yield components seed mineral content

All plants were harvested manually at physiological maturity. The number of seeds in each pod was collected and then weighed to obtain 100-grain weight (GW) for each treatment. The biological yield (BY) was determined by weighing the total plants in each pot and then the average was calculated. Similarly, grain yield (GY) was determined by weighing the total number of grains produced by plants per pot, and later average number of grains per plant was calculated.

Finely ground seeds (0.2 g) of each treatment were digested by HNO_3_ to determine the concentrations of mineral nutrients. The potassium (K), zinc (Zn), iron (Fe), and manganese (Mn) concentrations were measured following the method of Karpiuk et al. (2016) while the S content was determined according to Chandra and Pandey (2016) using atomic absorption spectrophotometry. Also, the seed N and P concentrations were measured on a UV–visible spectrophotometer (Hitachi-220, Hitachi Ltd, Tokyo, Japan) using Kjeldahl method (AOAC 1984) and molybdenum reaction solution method (Chen et al. 1956), respectively.

#### Statistical analyses

Fisher’s ANOVA (analysis of variance) technique was employed to statistically analyze all recorded data using Statistix (Analytical Software, FL, USA, Version 8.1). The treatment means were compared using Tukey’s *posthoc* HSD test at a 95% confidence level (*P* < 0.05).

## Results

### Biomass attributes

**Fig. 1:**
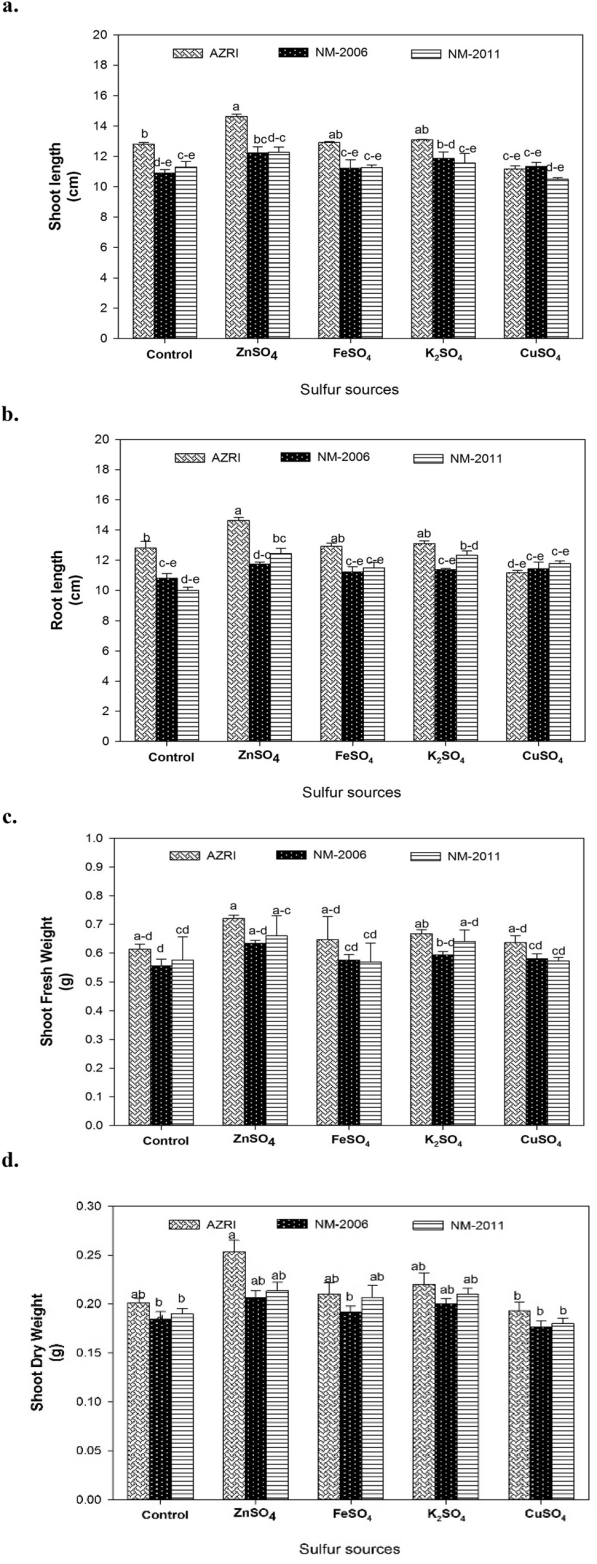
Effect of different S-sources viz. no S supply (control), ZnSO_4_, FeSO_4_, K_2_SO_4_ and CuSO_4_ on **a** shoot length, **b** root length **c** shoot fresh weight and **d** shoot dry weight of three mung bean cultivars (NM-2006, NM-2011 and AZRI-2006). Treatment means that are significantly different are represented by different letters, after applying *post-hoc* Tukey’s HSD test

All biomass traits exhibited highly considerable (*P* ≤ 0.01) variation among mung bean cultivars (Suppl. Table 1). The cultivar AZRI-2006 maintained significantly higher SL (12.64 cm), RL (12.12 cm), SFW (0.72 g), and SDW (0.27 g) compared to NM-2006 and NM-2011 (Fig. 1). Among different SO_4_^2−^ fertilizers, ZnSO_4_ application resulted in the highest SL (37%), RL (51%), SFW (26%), and SDW (25%) in AZRI-2006 with respect to control. Application of FeSO_4_ and K_2_SO_4_ also improved the biomass of AZRI-2006 seedlings only, whereas CuSO_4_ markedly reduced these attributes in all mung bean cultivars (Fig. 1).

### Shoot NPK content

**Fig. 2:**
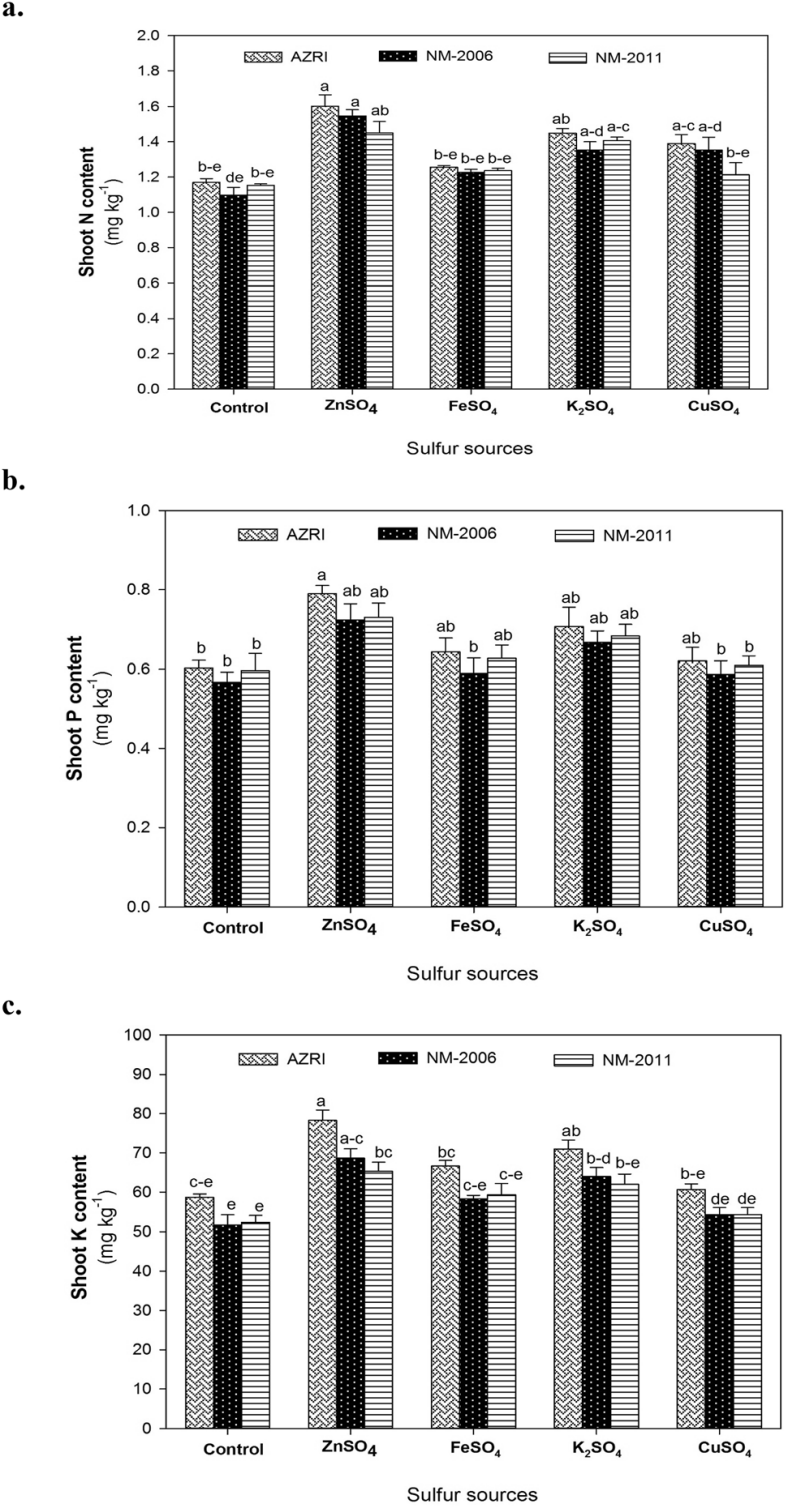
Effect of different S-sources viz. no S supply (control), ZnSO_4_, FeSO_4_, K_2_SO_4_ and CuSO_4_ on shoot **a** nitrogen, **b** phosphorous and **c** potassium content of three mung bean cultivars (NM-2006, NM-2011 and AZRI-2006). Treatment means that are significantly different are represented by different letters, after applying *post-hoc* Tukey’s HSD test

Among mung bean cultivars, a marked variation for shoot NPK content was only noted for K accumulation as AZRI-2006 exhibited 13 and 12% higher K content than NM-2006 and NM-2011, respectively (Fig. 2c). However, different S fertilizers showed a significant effect on N, P, and K accumulation in shoots of mung bean cultivars (*P* ≤ 0.01; Suppl. Table 1). Mung bean cultivars treated with ZnSO_4_ exhibited 25 and 9% higher shoot N content than FeSO_4_ and K_2_SO_4_, respectively (Fig. 2a). A similar trend was noted for shoot P content with a maximum increase (25%) recorded in shoots of seedlings fertigated with ZnSO_4_ compared to control (no S fertilizer). Likewise, the K_2_SO_4_ application also improved shoot P content by 16%, whereas no significant effect of FeSO_4_ and CuSO_4_ was observed on this variable (Fig. 2b). The shoot K content was increased by 30 and 21% in seedlings treated with ZnSO_4_ and K_2_SO_4_, respectively however, CuSO_4_ application did not affect K accumulation compared to control (Fig. 2c).

### Leaf RWC and SPAD value

**Fig. 3:**
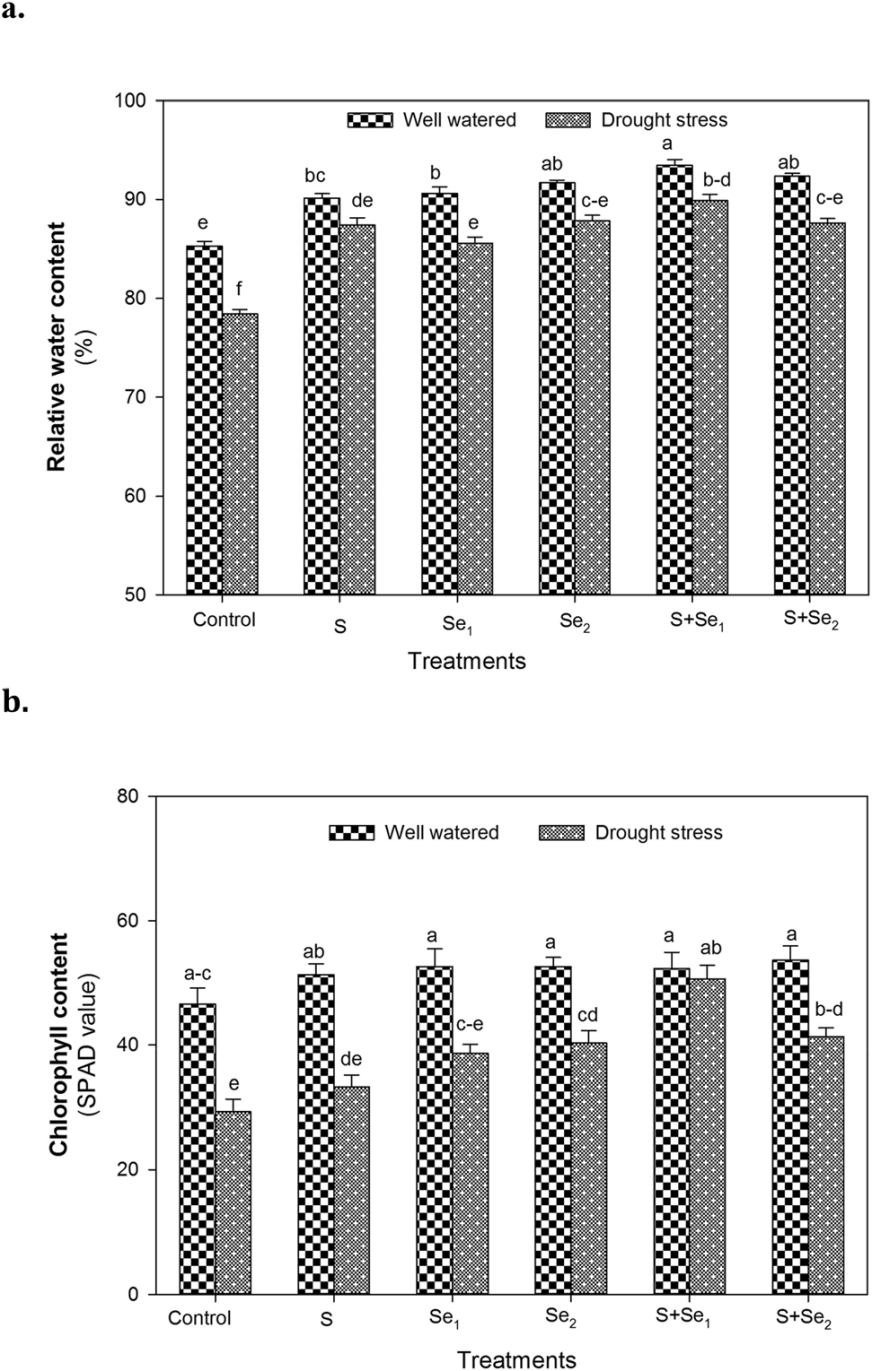
The individual and combined effect of S (ZnSO_4_) and Se (Na_2_SeO_4_) on **a** leaf relative water content and **b** total chlorophyll content of mung bean plants under well-watered and drought stress conditions. S_1_ = 120 mg S pot^−1^, Se_1_ = 2 μmol Se L^−1^, Se_2_ = 4 μmol Se L^−1^, S + Se_1_ = 120 mg S pot^−1^ + 2 μmol Se L^−1^ and S + Se_2_ = 120 mg S pot^−1^ + 4 μmol Se L^−1^. The significant differences among treatment means are represented by different letters, after applying *post-hoc* Tukey’s HSD test

Drought stress considerably decreased (*P* ≤ 0.01) the leaf RWC of mung bean plants (Suppl. Table 2). Under well-watered and drought conditions, the combined application of S and Se (S + Se_2_) resulted in maximum leaf RWC and improved it by 10 and 16% in normal and water-stressed plants, respectively compared to control. Individual application of ZnSO_4_ (S) and low (Se_1_) or high Se dose (Se_2_) also improved leaf RWC by 9 and 12%, respectively under drought stress conditions (Fig. 3a).

**Table 2:** The net photosynthetic rate (*A*), transpiration rate (*E*), stomatal conductance (*g*_*s*_) and sub-stomatal conductance (*C*_*i*_) of mung bean plants under well watered and drought stress conditions. The values indicate the treatment means of three replicates. The means with same alphabets exhibit no significant difference

Treatments	*A* (μmol CO_2_ m^−2^ s^−1^)	*E* (mmol H_2_O m^−2^ s^−1^)	*g*_*s*_ (mmol H_2_O m^−2^ s^−1^)	*C*_*i*_ (mmol H_2_O m^−2^ s^−1^)
*Well watered*				
Control	5.90^b^	3.26^d^	38.33^bc^	133.67^b−d^
S	6.0^ab^	3.73^bc^	46.67^ab^	151.0^a−c^
Se_1_	5.87^ab^	3.46^cd^	47.0^ab^	152.33^a−c^
Se_2_	6.83^ab^	3.65^cd^	51.67^ab^	166.0^a^
S + Se_1_	7.0^a^	4.15^a^	51.33^ab^	157.0^a−c^
S + Se_2_	6.77^ab^	4.14^ab^	58.0^a^	159.67^ab^
*Drought stress*				
Control	3.43^d^	0.87^g^	13.67^e^	61.67^g^
S	4.47^cd^	1.09^fg^	17.0^de^	81.33^fg^
Se_1_	4.0^cd^	1.01^g^	19.0^de^	76.0^fg^
Se_2_	4.37^cd^	1.45^ef^	27.67^c−e^	94.0^ef^
S + Se_1_	4.60^c^	1.60^e^	25.0^c−e^	114.33^de^
S + Se_2_	4.80^c^	1.65^e^	29.33^cd^	125.67^cd^
Tukey HSD_0.05_	1.06	0.41	14.31	31.64
CV	6.73	5.56	13.63	8.69

The exposure to drought showed a marked reduction (32%) in leaf Chl content (measured as a SPAD value) of mung bean plants (*P* ≤ 0.01, Suppl. Table 2). Under well-watered conditions, no significant effect of individual or combined S and Se doses was observed on this variable, whereas S + Se_1_ markedly enhanced leaf Chl (72%) under drought stress conditions (Fig. 3b). The leaf Chl were also increased by individual Se application as low (Se_1_) or high Se dose (Se_2_) improved SPAD value by 38 and 34%, respectively while ZnSO_4_ supply alone (S) did not affect leaf Chl compared to control under water deficit conditions (Fig. 3b).

### Gas exchange

A significant variation (*P* ≤ 0.01) existed among individual or combined treatment of S and Se for leaf gas exchange attributes under well-watered and drought stress conditions (Suppl. Table 2). Under well-watered conditions, the highest increase (32%) in leaf *A* was observed in plants treated with S in combination with 2 μmol Se L^−1^ i.e. S + Se_1_ however, it resulted in a much higher increase (80%) in leaf *A* statistically related to S + Se_2_ (120 mg S pot^−1^ + 4 μmol Se L^−1^) compared to control (no S and/or Se application) under drought stress (Table 2). In the plants exposed to drought stress, leaf *g*_*s*_ and *C*_*i*_ were increased by 87 and 92%, respectively by S + Se_2_ application, in contrast to 62 and 23% increase in these variables compared to control under normal conditions (Table 2). Similarly, the highest leaf *E* was also maintained by the plants treated with combined S + Se_2_ application (88%) statistically at par with S + Se_1_ (85%) with respect to control under drought stress conditions (Table 2).

### Enzyme antioxidants

**Fig. 4:**
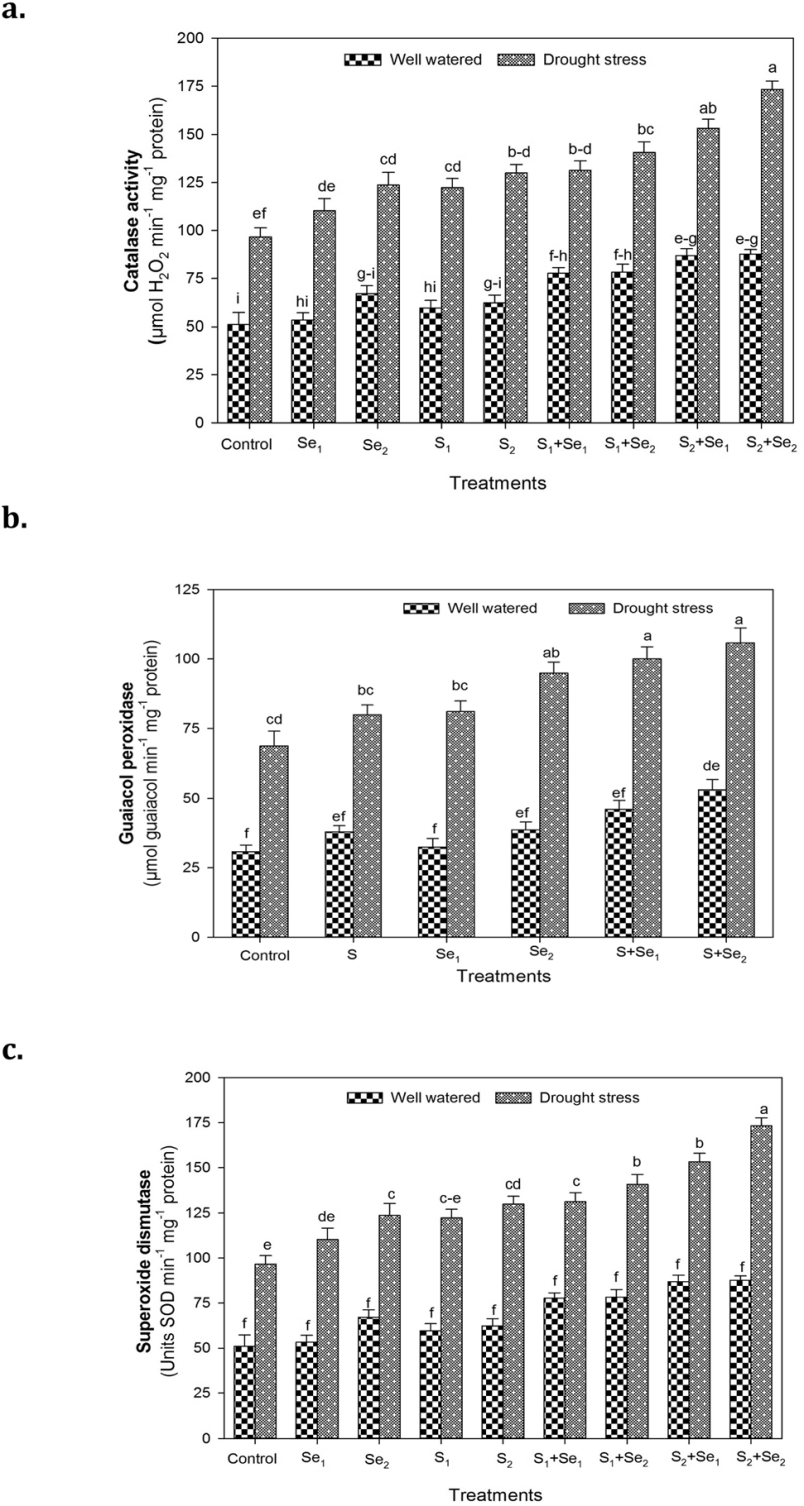
The individual and combined effect of S (ZnSO_4_) and Se (Na_2_SeO_4_) on enzyme activity of **a** catalase **b** guaiacol peroxidase and **c** superoxide dismutase in leaves of mung bean plants under well-watered and drought stress conditions. S_1_ = 120 mg S pot^−1^, Se_1_ = 2 μmol Se L^−1^, Se_2_ = 4 μmol Se L^−1^, S + Se_1_ = 120 mg S pot^−1^ + 2 μmol Se L^−1^ and S + Se_2_ = 120 mg S pot^−1^ + 4 μmol Se L^−1^. The significant differences among treatment means are represented by different letters, after applying *post-hoc* Tukey’s HSD test

Drought stress markedly enhanced the activities of enzyme antioxidants viz. CAT, GPX, and SOD in the leaves of mung bean plants, irrespective of S and Se treatments (Suppl. Table 2). The highest increase in activity of antioxidative enzymes was recorded in the combination of S and Se treatments i.e. S + Se_1_ and S + Se_2_ (Fig. 4). Mung bean plants treated with S + Se_2_ exhibited the highest CAT (79%) and GPX (53%) activity statistically related to S + Se_1_ treatment with respect to control under drought stress. Among individual treatments, foliar spray of high Se dose (Se_2_) also stimulated CAT (28%) and GPX (37%) activities, whereas low Se dose (Se_1_) or S application enhanced CAT activity by 17 and 26% (Fig. 4a) and GPX activity by 19% (Fig. 4b) compared to control under water deficit conditions. The highest increase in SOD activity (81%) was also recorded in water-stressed mung bean plants applied with combined S + Se_2_ treatment (Fig. 4c). No significant variation for SOD activity was recorded between S + Se_1_ and Se_2_ treatments that stimulated enzyme activity by 58%, whereas Se_1_ and S did not considerably affect SOD activity with respect to control under water deficit conditions.

### Yield attributes

**Fig. 5:**
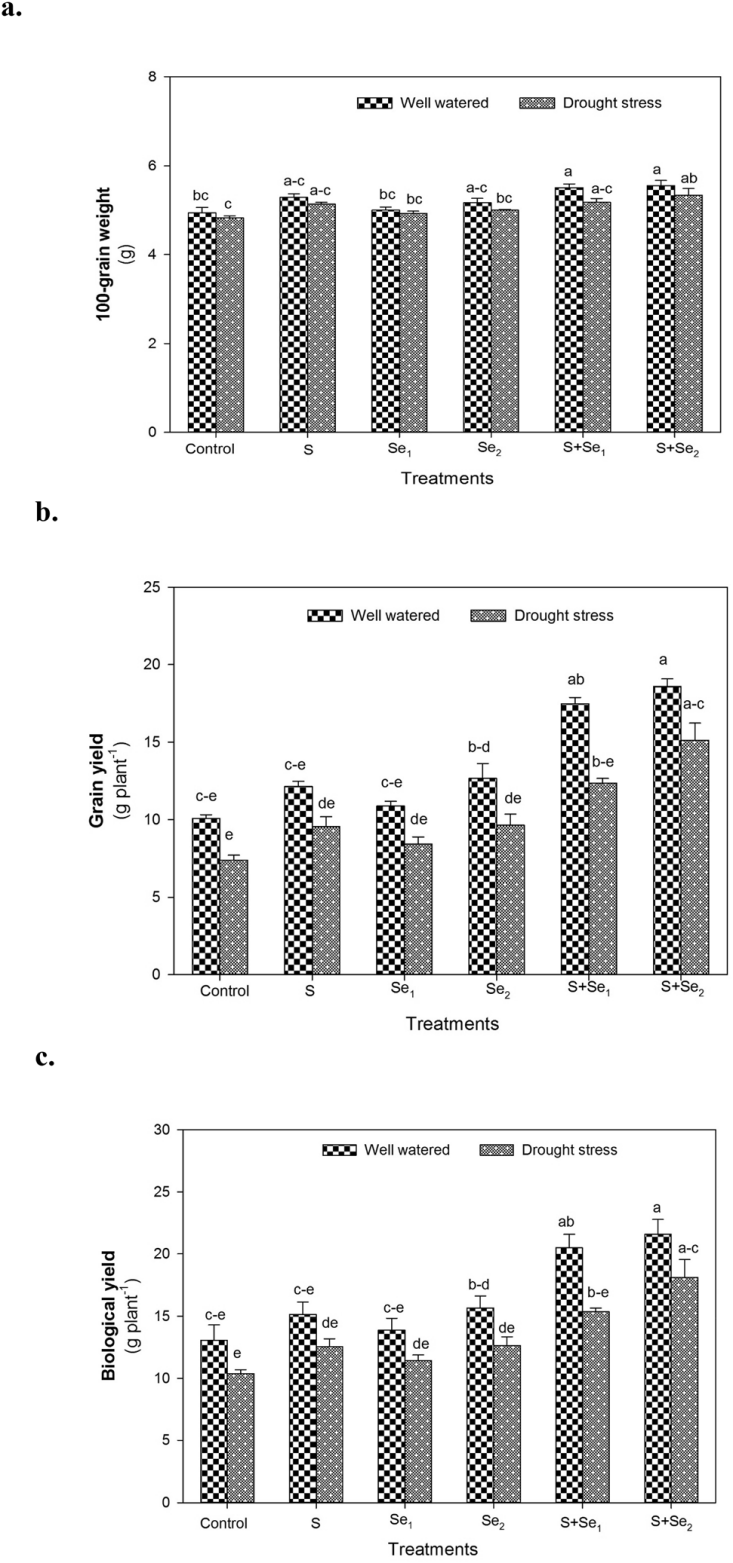
The individual and combined effect of S (ZnSO_4_) and Se (Na_2_SeO_4_) on **a** 100-grain weight **b** grain yield and **c** biological yield of mung bean plants under well-watered and drought stress conditions. S_1_ = 120 mg S pot^−1^, Se_1_ = 2 μmol Se L^−1^, Se_2_ = 4 μmol Se L^−1^, S + Se_1_ = 120 mg S pot^−1^ + 2 μmol Se L^−1^ and S + Se_2_ = 120 mg S pot^−1^ + 4 μmol Se L^−1^. The significant differences among treatment means are represented by different letters, after applying *post-hoc* Tukey’s HSD test

Drought stress, in relation to well-watered conditions, caused a marked reduction in mung bean yield (*P* ≤ 0.01, Suppl. Table 3). Mung bean plants treated with S and Se in combination (S + Se_1_, S + Se_2_) showed marked improvement in GW, GY and BY under both well-watered and drought stress conditions. In comparison to no S or Se application (control), combined S + Se_2_ application resulted in the highest increase in GW (11%), GY (105%), and BY (75%) followed by S + Se_1_ treatment that improved GW, GY, and BY by 7, 68 and 48%, respectively under water deficit conditions, whereas no significant differences were obtained when S or Se was applied as individual treatments (Fig. 5).

**Table 3:** The concentrations of nitrogen (N), phosphorous (P), potassium (K), zinc (Zn), sulfur (S), iron (Fe) and manganese (Mn) in the seeds of mung bean plants at maturity under well watered and drought stress conditions. The values indicate the treatment means of three replicates. The means with same alphabets exhibit no significant difference

Treatments	N (μg g^−1^ DW)	P (μg g^−1^ DW)	K (μg g^−1^ DW)	Zn (μg g^−1^ DW)	S (μg g^−1^ DW)	Fe (μg g^−1^ DW)	Mn (μg g^−1^ DW)
*Well watered*							
Control	1.21^de^	0.60^a^	5.87^a^	29.87^a^	0.38^ab^	61.90^a−c^	15.87^ab^
S	1.31^a−e^	0.65^a^	6.43^a^	34.73^a^	0.44^a^	70.23^ab^	17.40^ab^
Se_1_	1.27^b−e^	0.63^a^	6.33^a^	34.37^a^	0.42^ab^	64.97^a−c^	17.90^ab^
Se_2_	1.38^ab^	0.63^a^	6.47^a^	35.23^a^	0.43^ab^	66.43^a−c^	15.23^ab^
S + Se_1_	1.33^a−d^	0.66^a^	6.57^a^	33.20^a^	0.45^a^	71.07^a^	20.20^a^
S + Se_2_	1.40^a^	0.63^a^	6.63^a^	35.83^a^	0.46^a^	71.30^a^	18.73^ab^
*Drought stress*							
Control	1.20^e^	0.32^c^	3.03^e^	11.77^c^	0.17^d^	57.83^c^	12.90^b^
S	1.28^a−e^	0.39^bc^	3.73^de^	15.56^c^	0.22^cd^	61.50^a−c^	17.17^ab^
Se_1_	1.24^c−e^	0.34^c^	4.77^c^	18.11^c^	0.20^cd^	58.40^bc^	15.13^ab^
Se_2_	1.33^a−d^	0.39^bc^	4.57^cd^	19.63^bc^	0.16^d^	58.18^bc^	15.23^ab^
S + Se_1_	1.31^a−e^	0.47^b^	5.30^bc^	30.67^a^	0.31^bc^	62.43^a−c^	15.43^ab^
S + Se_2_	1.34^a−c^	0.38^b^	4.40^cd^	27.71^ab^	0.24^cd^	66.60^a−c^	17.73^ab^
Tukey HSD_0.05_	0.13	0.11	0.98	8.17	0.12	12.11	6.07
CV	3.27	7.35	6.16	10.11	12.83	6.35	12.34

### Seed mineral content

Under drought stress, the nutrient content (N, P, K, Zn, S, and Fe) in seeds of mung bean plants was considerably decreased (Suppl. Table 3). Particularly, seed K content was decreased by 48%, whereas P, Zn, and S concentrations in seeds were reduced by 65, 68 and 96%, respectively compared to seeds of plants grown under well-watered conditions (Table 3). Seeds from mung bean plants grown with S, Se or S + Se showed considerable enhancement in mineral content. As compared to no S or Se supply (control), the highest increase in seed concentrations of P (47%) and K (75%) was recorded by S + Se_1_ (Table 3)_,_ whereas S + Se_2_ resulted in the maximum seed N content (12%) closely followed by Se_2_ (11%) and S + Se_1_ (10%) under drought stress conditions (Table 3). Although differences in S and Zn seed concentrations between the individual or combined S or Se treatments were not significant under well-watered conditions, a marked enhancement in seed S (80%) and Zn (160%) content was recorded by S + Se_1_ treatment compared to control under water deficit conditions (Table 3). Similarly, S + Se_2_ and Se_2_ improved Zn seed concentrations by 125 and 67%, respectively while no application (control) gave the lowest values for Zn seed concentrations followed by individual S or low Se supply (Se_1_) treatments in water-stressed mung bean plants (Table 3). Under well-watered conditions, seed Fe and Mn concentrations were significantly increased by 15 and 26%, respectively in plants treated with combined S and Se applications i.e. S + Se_1_ and S + Se_2_ compared to control. A similar trend was noted in water-stressed plants as S + Se_2_ resulted in the highest (15%) seed Fe content (Table 3), however, a much higher increase of 37% was noted for Mn concentration by this treatment compared to control (Table 3). In contrast, individual S and Se doses were found statistically similar to control regarding seed Fe and Mn content.

## Discussion

Most agricultural soils lack S and contain less than 10% of the total soil S in the plant available form i.e. SO_4_ (Urton et al. 2018). Consequently, the organic S sources (soil organic matter and crop residues) are primary SO_4_ sources for plants during the growth season (Havlin et al. 2014). The increase in biomass of mung bean cultivars in response to different S fertilizers compared to control (no S supply) is consistent with the availability of low SO_4_ levels in soil used for these experiments. A marked improvement in biomass was noted by S fertilizer amendments, which may be due to the positive effects of S on cell division resulting in leaf expansion and light absorption that ultimately improved the vegetative growth (Garg et al. 2006). A highly significant increase in SL was noted in plants supplemented with various SO_4_ fertilizers except CuSO_4_ (Fig. 1a). Similarly, a considerable increase in RL and dry matter content was also observed in mung bean plants treated with S fertilizers (Fig. 1). Availability of SO_4_ in growth medium is positively correlated to indole-3-acetic acid (IAA) levels in root meristem that promotes primary root elongation to facilitate the uptake of nutrients and moisture (Zhao et al. 2014). A positive effect of S fertilizers on the dry matter of mung bean plants might be due to increased carbohydrate utilization for protein synthesis in the presence of S (Shekhawat and Shivay 2012). These results are in line with studies on wheat (Salvagiotti and Miralles 2008) and barley (De Bona et al. 2011) grown on SO_4_ deficient soils. Among various S fertilizers, the maximum biomass was attained in plants fertilized with ZnSO_4_. It might be attributed to regulatory functions of both Zn^2+^ and SO_4_^2−^ to stimulate the activity of enzymes such as aldolases, hydrogenases, nucleic acid polymerases. Moreover, they also act as a cofactor to regulate protein synthesis thereby contributing to increased biomass in plants (Rossi et al. 2018). The possible explanation for the decrease in biomass by CuSO_4_ application may be the presence of Cu^2+^ that alters root morphology to decrease xylem loading of water and nutrients in plants (Chen et al. 2015).

The metabolism of N in plants is strongly affected by the S status of the plant (Anjum et al. 2012). It was observed that NPK uptake was considerably higher in AZRI-2006 compared to NM-2006 and NM-2011 (Fig. 2). These results suggest that AZRI-2006 has a high capacity to uptake NPK and can respond better to SO_4_^2−^ availability. Our results are in agreement with reports of Lee et al. (2015) who observed significant variation among *Brassica* species in their response to S nutrition. Among S-fertilizers, ZnSO_4_ and FeSO_4_ markedly improved N uptake in mung bean plants (Fig. 2a). The increased N content by ZnSO_4_ might be related to increased activity of SO_4_^2−^ transporters in the root with Zn^2+^ exposure resulting in enhanced NO_3_^−^ absorption and translocation in leaves (Stuiver et al. 2014). Plants have well-developed S and N pathways, which are functionally convergent as the availability of one nutrient regulates the other (Kaur et al. 2013). A marked increment in P uptake was observed in plants treated with S-fertilizers. Taalab et al. (2008) reported that combined application of different P sources with S results in better uptake of macronutrients such as N, P, and K compared to P alone. Likewise, S-application enhanced K content in the shoot, which might be associated with the fact that S and K act as counter-ions (Bäucker et al. 2003). Chinese cabbage plants exposed to S deficiency were observed to accumulate fewer K^+^ in the shoot by Reich et al. (2016). Increased NPK accumulation in mung bean leaves by FeSO_4_ application provides further evidence regarding the cooperative effect of Fe-S clusters on plant metabolism (Lu 2018). In contrast, Cu^2+^ exposure in the root zone markedly decreases the uptake and distribution of SO_4_^2−^ (Shahbaz et al. 2014) that also reduces NPK uptake due to the limited incorporation of proteins in leaves.

Mung bean is considered a drought-tolerant crop, however; water deficiency at critical growth stages may result in significant yield losses (Singh and Singh 2011). Application of S has been observed as increasing the resistance of plants under drought stress as it helps to regulate metabolic processes and is the main constituent of metabolites involved in protein synthesis under water-limited conditions (Vazin 2012). Foliar treatment of mung bean plants with Se in combination with S (S + Se) considerably improved RWC (Fig. 2a), which signifies synergistic relation of these nutrients in the accumulation of solutes to sustain water status under drought (Hartikainen 2005). Se increases water retention in the plant tissues through improved uptake of water by the root system (Nawaz et al. 2015a). The regulation of stomatal apparatus with S + Se supply (as observed in the present study, Table 2) might be responsible for improved leaf RWC under water stress conditions (Usmani et al. 2020). The hydraulic conductance of plant species is a major function for increased water status under stress conditions (Schultz 2003) hence, an increase in RWC of plants treated with S + Se indicates the positive role of these nutrients in increasing water uptake by roots without decreasing *E* under water deficit conditions. S availability helps to maintain osmotic potential by restricting the cellular accumulation of ions and solutes (Lee et al. 2015). Similarly, Se helps to maintain membrane integrity (Chauhan et al. 2019) or reduces oxidative damage (Shekari et al. 2019). Photosynthetic pigments like chlorophyll indicate the photosynthetic capacity of plants (Chutipaijit et al. 2011). It is well reported that exogenous S or Se supply prevents the damage to chloroplasts and increases the pigments under drought stress conditions (Usmani et al. 2020). A marked increase in SPAD value (an indicator of total leaf Chl) was noted with S + Se supply which might be associated with sulphurs’ role in the maintenance of chloroplast targeted proteins (cysteine and methionine) as well as Se mediated scavenging of ROS through increased activity of antioxidants (also observed in the present study) to inhibit lipid peroxidation that leads to chlorophyll destruction (Habibi 2013).

As expected, low water availability markedly reduced *A*, *E, g*_*s*_*,* and *C*_*i*_ in leaves of mung bean plants. The reduction in *A* result from decreased *g*_*s*_ that restricts CO_2_ assimilation and uptake of nutrients from the soil due to limited *E*. In this study, the application of S + Se considerably improved *A* (Table 2) suggesting these nutrients work synergistically to prevent Rubisco deactivation, chlorophyll degradation, and stomatal closure. This might also be attributed to the fact that both S and Se positively influence turgor maintenance, thereby increasing tolerance in a water-limited environment. S deficiency has been reported to severely restrict *A* in *Brassica napus* (Muneer et al. 2014), *Brassica juncea* (Fatma et al. 2014), and *Hordeum vulgare* (Astolfi et al. 2012) indicating that S is crucial to maintain *A* in plants. S is a vital constituent of ferredoxin and lipids found in the chloroplast. Moreover, sulfur bonds (-S–S-) are very crucial for maintaining protein structure and disulfide groups are constituents of active sites of many enzymes (Speiser et al. 2018). Application of Se and S increased the *A*, *E,* and *g*_*s*_, however, maximum activity was observed at combined higher doses of Se and S. This may be due to the synergistic effects of S and Se as both these nutrients share a similar assimilation pathway (Sors et al. 2005). Se-mediated increase in *A*, *E,* and *g*_*s*_ has also been reported by Nawaz et al. (2015a) in wheat and Proietti et al. (2013) in olive plants. Moreover, Djanaguiraman et al. (2010) showed that Se-spray elevated *A* in sorghum that might be related to Se-mediated low ROS levels and enhanced antioxidant activities.

A marked increment in enzyme activities of antioxidants was recorded by the application of S and Se in water-stressed mung bean plants, which could be attributed to the protective role of these enzymes to detoxify the elevated H_2_O_2_ into water and oxygen (Anjum et al. 2012). Enhanced enzyme activity of antioxidants in mung bean supplemented with exogenous Se has also been reported earlier (Malik et al. 2012; Alam et al. 2019). A well-organized antioxidant system plays a critical role to scavenge ROS under environmental stress conditions (Karl et al. 2009). The observed increase in SOD activity of mung bean plants by combined application of S (120 mg pot^−1^) and Se (4 μmol L^−1^) under drought stress (Fig. 4c) provides further evidence about the protective characteristics of S and Se to attenuate damaging effects of ROS and regulate the osmotic status of plants (Hajiboland et al. 2015). Plants produce SOD as a primary defense enzyme to scavenge ROS produced under drought (Hussain et al. 2016). Turkan et al. (2005) reported higher activity of CAT in drought-tolerant varieties of bean cultivars compared to sensitive ones. Plants supplemented with S + Se showed higher GPX and CAT activity and increased tolerance to the water-limited conditions. Increased GPX activity by Se supplementation corresponds to the reports of Yao et al. (2012). It may be inferred that high GPX activity promotes lignin biosynthesis that contributes to enhanced cell wall rigidity and increased uptake of mineral nutrients, thereby regulating the defense mechanism of plants to scavenge H_2_O_2_ (Gill and Tuteja 2010; Majeed et al. 2020).

Drought-induced significant reduction in mung bean yield was recorded in the present study (Fig. 5). The reduction in yield mainly results from a decreased translocation of nutrients due to the short duration of seed filling induced by drought stress (Nair et al. 2019). Moreover, water deficiency at the flowering stage causes flower and pod abortion resulting in small-sized seeds (Fang et al. 2010). In the present study, a significant increase in GW was recorded with combined S and Se application. S-mediated enhancement in yield has been reported in crops such as chickpea (Abd Allah et al. 2015), maize (Usmani et al. 2020), wheat (Tabak et al. 2019), and mung bean (Sachan et al. 2019). It has been suggested that S supply in combination with Se increases nutrient uptake particularly N and its translocation for the incorporation of proteins in seeds (Becana et al. 2018). Moreover, S + Se regulated enhanced photosynthetic activity also positively influenced the GW. A marked increase in BY was recorded by combined S + Se application (Fig. 5b). It is more likely the consequence of efficient translocation of assimilates resulting in increased dry matter thereby increasing BY under water-deficient conditions. The increase in GY by S + Se could be ascribed to improved water status (Fig. 2a) and gas exchange characteristics (Table 2), thereby improving GY under water deficit conditions.

The concentrations of minerals in grains are regulated by direct uptake from soil and/or remobilization of nutrient reserves in tissues during grain filling (Kutman et al. 2011). Exposure to drought impairs root architecture resulting in reduced nutrient uptake and changes in quantity of nutrients accumulated during seed filling (Sehgal et al. 2018). In this study, fewer negative effects of drought were noted on the nutrient content of mung bean seeds obtained from plants treated with the combined application of S and Se. Limited water availability restricts SO_4_^2−^ uptake and its translocation to leaves (Usmani et al. 2020). Moreover, it also reduces nitrogen use efficiency (NUE) due to decreased NO_3_^−^ absorption under water deficit conditions (Lee et al. 2009). Drought stress decreases protein incorporation in leaves due to low S demand that significantly limits SO_4_^2−^ uptake in water-stressed plants (Anderson and Fitzgerald 2003). Previous studies showed marked increment in S accumulation by Se application in lettuce (Ramos et al. 2011) *Arabidopsis* (White et al. 2004) and Kale (Lefsrud et al. 2006). Similarly in the present study, NPK content in seeds was significantly enhanced by S + Se application in water-stressed plants (Table 3). Foliar Se spray not only increased N, P, and K concentrations but also enhanced Zn, S, Fe, and Mn content in mung bean seeds. Similarly, Reis et al. (2018) reported positive interaction between Se and N that significantly increased the quality of rice seeds. In contrast, a negative correlation was recorded between Se and N in barley (Ilbas et al. 2012), which may be due to application of high Se doses resulting in Se toxicity. Similar to our observations, Eich-Greatorex et al. (2010) observed high P uptake by Se application suggesting that Se interactions with phosphate are largely dependent on the source of Se as selenite uptake is not efficient in P-enriched medium (Dinh et al. 2019). Contrary to our earlier report about no significant effect of Se on K content of wheat seeds (Nawaz et al. 2015b), an increased K content was recorded in mung bean seeds with S + Se treatment. Likewise, S content were also improved by combined S and Se application supporting the observations of Guerrero et al. (2014) who observed that selenate promotes translocation of S and noted increased shoot S content in wheat. There was an increase in seed Zn content by S + Se application, which may likely be the consequence of the application of ZnSO_4_ as a source of S. However, Se application combined with S resulted in high Zn content compared to individual treatment of S or Se (Table 3). These observations correspond to the reports of de Figueiredo (2017) who observed that common bean plants treated with Se or Zn maintained a constant level of these minerals during seed development. An increased seed Fe and Mn content by S + Se application could partly be ascribed to positive interactions of Fe with S and Se that favors the formation of Fe-S clusters in cells involved in the photosynthetic activity (Nazar et al. 2011) thereby, alleviating negative effects of drought stress on uptake and translocation of mineral nutrients.

## Conclusion

In conclusion, the combined application of S and Se (S + Se) was observed to confer drought tolerance in mung bean, by alleviating the damage caused by drought stress and stimulating the physiological processes such as increased water status, chlorophyll content and photosynthetic activity. Moreover, the increased activity of antioxidative enzymes by S + Se application mitigated water deficit stress and improved yield and mineral status of mung bean under drought. These results could benefit farmers to obtain high nutrient concentrations in mung bean seeds with a limited supply of water. Besides, these results may also be of great interest to researchers including plant breeders to identify candidate genes regulated by both S and Se, which play an important role in improving the drought tolerance and controlling mineral accumulation in seeds.

## Supplementary Information

Below is the link to the electronic supplementary material.

Supplementary file1 (DOCX 66 kb)
