# Supplementary material for: Physiological insights into sulfate and selenium interaction to improve drought tolerance in mung bean
Source: Physiol Mol Biol Plants. 2021 May 4;27(5):1073–87. doi: 10.1007/s12298-021-00992-6 (PMC8140040; doi:10.1007/s12298-021-00992-6)
Supplement: Supplementary file 1 — Supplementary file1 (DOCX 66 kb) [file 12298_2021_992_MOESM1_ESM.docx]

**Suppl. Table 1:** Summary of the ANOVA shoot length (SL), root length (RL), shoot fresh weight (SFW), shoot dry weight (SDW), shoot nitrogen (N), phosphorous (P) and potassium (K) content of mung bean plants under well watered and drought stress conditions.

| **SOV*** | **SL**  **(cm)** | **RL**  **(cm)** | **SFW**  **(g)** | **SDW**  **(g)** | **Shoot N**  **(mg kg^-1^ DW)** | **Shoot P**  **(mg kg^-1^ DW)** | **Shoot K**  **(mg kg^-1^ DW)** |
| --- | --- | --- | --- | --- | --- | --- | --- |
| **Mung bean cultivars (V)** | *P*≤ 0.001 | *P*≤ 0.001 | *P*≤ 0.001 | *P*≤ 0.01 | *P*≤0.05 | *P*≤ 0.05 | *P*≤ 0.001 |
| **Sulfur sources (S)** | *P*≤ 0.001 | *P*≤ 0.001 | *P*≤ 0.001 | *P*≤0.01 | *P*≤ 0.01 | *P*≤ 0.001 | *P*≤ 0.001 |
| **V × S** | *P*≤0.001 | NS | NS | NS | *P*≤0.05 | NS | NS |
| **CV** | 4.14 | 4.20 | 5.05 | 9.25 | 7.71 | 8.95 | 6.64 |

**Suppl. Table 2:** Summary of the ANOVA for leaf relative water content (RWC), chlorophyll content (Chl), photosynthetic rate (*A*), transpiration rate (*E*), stomatal conductance (*g_s_*), sub-stomatal conductance (*C_i_*), catalase (CAT), guaiacol peroxidase (GPX) and superoxide dismutase (SOD) activity mung bean plants under well watered and drought stress conditions.

| **SOV** | **RWC**  (%) | **Chl**  (SPAD value) | ***A***  (µmol CO_2_ m^-2^ s^-1^) | ***E***  (mmol H_2_O m^-2^ s^-1^) | ***g_s_***  (mmol H_2_O m^-2^ s^-1^ | ***C_i_***  (µmol H_2_O m^-2^ s^-1^) | **CAT**  (µmol H_2_O_2_ min^-1^ mg^-1^ protein) | **GPX**  (µmol guaiacol min^-1^ mg^-1^ protein) | **SOD**  (Units SOD min^-1^ mg^-1^ protein) |
| --- | --- | --- | --- | --- | --- | --- | --- | --- | --- |
| Drought stress (D) | *P*≤ 0.001 | *P*≤ 0.001 | *P*≤ 0.001 | *P*≤ 0.001 | *P*≤ 0.001 | *P*≤ 0.001 | *P*≤ 0.001 | *P*≤ 0.001 | *P*≤ 0.001 |
| Fertilzer treatments (F) | *P*≤ 0.001 | *P*≤ 0.001 | *P*≤ 0.001 | *P*≤ 0.001 | *P*≤ 0.001 | *P*≤ 0.001 | *P*≤ 0.001 | *P*≤ 0.001 | *P*≤ 0.001 |
| D × F | *P*≤ 0.05 | *P*≤ 0.01 | NS | NS | NS | *P*≤ 0.01 | NS | NS | *P*≤ 0.001 |
| CV | 1.05 | 7.61 | 6.73 | 5.56 | 13.63 | 8.69 | 9.68 | 9.87 | 10.71 |

**Suppl. Table 3:** Summary of the ANOVA for 100-grain weight (GW), grain yield (GY), biological yield (BY), seed nitrogen (N), phosphorous (P), potassium (K), zinc (Zn), sulfur (S) and manganese (Mn) content of mung bean plants under well watered and drought stress conditions.

| **SOV** | **GW**  (g) | **GY**  (g plant^-1^) | **BY**  (g plant^-1^) | **N**  (μg g^-1^ DW) | **P**  (μg g^-1^ DW) | **K**  (μg g^-1^ DW) | **Zn**  (μg g^-1^ DW) | **S**  (μg g^-1^ DW) | **Fe**  (μg g^-1^ DW) | **Mn**  (μg g^-1^ DW) |
| --- | --- | --- | --- | --- | --- | --- | --- | --- | --- | --- |
| Drought stress (D) | *P*≤ 0.01 | *P*≤ 0.001 | *P*≤ 0.001 | *P*≤ 0.001 | *P*≤ 0.001 | *P*≤ 0.001 | *P*≤ 0.001 | *P*≤ 0.001 | *P*≤ 0.001 | *P*≤ 0.05 |
| Fertilzer treatments (F) | *P*≤ 0.001 | *P*≤ 0.001 | *P*≤ 0.001 | *P*≤ 0.05 | *P*≤ 0.01 | *P*≤ 0.001 | *P*≤ 0.001 | *P*≤ 0.01 | *P*≤ 0.001 | *P*≤ 0.01 |
| D × F | NS | NS | NS | NS | NS | *P*≤ 0.01 | *P*≤ 0.001 | NS | NS | NS |
| CV | 3.15 | 14.41 | 11.54 | 3.27 | 7.35 | 6.16 | 10.11 | 12.83 | 6.35 | 12.34 |
